# Graded prioritisation of targets in search: reward diminishes the low prevalence effect

**DOI:** 10.1186/s41235-023-00507-9

**Published:** 2023-08-04

**Authors:** Veronica Hadjipanayi, Casimir J. H. Ludwig, Christopher Kent

**Affiliations:** University of Bristol, Bristol, UK

**Keywords:** Prevalence effect, Reward, Multiple target search, Visual search

## Abstract

In many real-life contexts, observers are required to search for targets that are rarely present (e.g. tumours in X-rays; dangerous items in airport security screenings). Despite the rarity of these items, they are of enormous importance for the health and safety of the public, yet they are easily missed during visual search. This is referred to as the prevalence effect. In the current series of experiments, we investigate whether unequal reward can modulate the prevalence effect, in a *multiple* target search task. Having first established the impact of prevalence (Experiment 1) and reward (Experiment 2) on how efficiently participants can find one of several targets in the current paradigm, we then combined the two forms of priority to investigate their interaction. An *unequal* reward distribution (where lower prevalence items are more rewarded; Experiment 3) was found to diminish the effect of prevalence, compared to an *equal* reward distribution (Experiment 4) as indicated by faster response times and fewer misses. These findings suggest that when combined with an unequal reward distribution, the low prevalence effect can be diminished.

## Public significance statement

This study highlights peoples’ ability to prioritise search for some targets over others based on their assigned priority (i.e. reward or prevalence). When these two types of priority were in conflict, results indicated a stronger effect of prevalence over reward on peoples’ visual search performance. Two different types of reward distribution were used, equal and unequal, with findings suggesting a different impact of each one on the prevalence effect. An unequal reward distribution improved peoples’ ability to quickly and accurately detect low prevalence targets, compared to an equal reward distribution. These results further our understanding of the cognitive aspects of the low prevalence effect which constitutes of a critical problem in real-life tasks with important consequences to the health and the security of the public (e.g. airport security screening, medical X-rays).

## Introduction

Visual search for a target amongst distractors is a critical component of everyday life. Whether we are searching for a friend in the crowd or for a specific product in a supermarket aisle, we are constantly required to search for behaviourally relevant information among an immense stream of less relevant visual input. There are often cases when we are searching for relatively rare targets which can be a challenging process. Take, for example, airport security X-rays for potentially dangerous objects in luggage (e.g. guns, knives, explosive devices) and medical X-ray screening tasks for tumours (e.g. mammography) or cytopathology screening (‘Pap tests’). The likelihood of finding a dangerous item in airport security screenings is relatively low (Rubinstein, 2001), while only around 1% of medical X-ray cases involve a tumour (Breast Cancer Surveillance Consortium, 2009; Fenton et al., 2007; Gur et al., 2003, 2004; Smith & Turnbull, 1997). While these events are rare, they are extremely important for security and health. The rare occurrence of such serious items decreases the efficiency with which they can be detected. This is referred to as the prevalence effect, according to which observers detect more quickly and efficiently objects that appear more often (high prevalence) in their visual field over objects that appear less often (low prevalence; Gur et al., 2003, 2004; Mitroff & Biggs, 2014; Schwark et al., 2012; Wolfe et al., 2005, 2007).

Wolfe et al. (2005) conducted an artificial baggage screening task where participants were presented with ‘tools’ as targets on a noisy background, at different levels of prevalence (i.e. 1%, 10%, 50%). They showed that participants often miss targets that are rarely present, but that the probability of such ‘miss errors’ decreased as target prevalence increased (Wolfe et al., 2005, 2007). This method of varying relative prevalence of targets during visual search has been used by other researchers as well (e.g. Godwin et al., 2010; Hout et al., 2015; Papesh et al., 2021; Walenchok et al., 2020). For instance, Godwin et al. (2015) monitored the eye movements of participants while they performed search with low prevalence (25% target-present trials), medium-prevalence (50%) or high-prevalence (75%) targets. As target prevalence increased, participants spent longer looking and revisiting the target. This indicates that as target prevalence increased, participants devoted more attention to that target during visual search. Hout et al. (2015) manipulated relative target prevalence in visual search and found that the impact of low prevalence still occurred even when motor errors were highly unlikely and early search termination errors were impossible, suggesting that the low prevalence effect is probably a result of perceptual errors.

Different explanations have been offered for the origin of ‘miss’ errors during visual search for low prevalence targets. Hon and Tan (2013) suggest that there are asymmetrical attentional demands between targets of different prevalence, with low prevalence targets being more attentionally demanding than high prevalence targets. That is, the authors argue that the establishment and maintenance of a template of a low prevalence target, which does not often appear in the visual field, are more cognitively demanding than that of a high prevalence target. As a result, high prevalence target templates are prioritised over low prevalence target templates, leading to reduced response time and miss errors for the former versus the latter. Alternatively, Fleck and Mitroff (2007) and Li et al. (2006) have proposed that participants in a visual search task similar to Wolfe et al. (2005) could decrease the amount of ‘miss’ errors during search for low prevalence targets if observers could slow down their responding or were permitted to correct their errors. This proposal implies that such correctable errors could be a result of a wrong motor act on behalf of participants (e.g. a “finger error” or lapse) or of a premature target-absent decision (driven by insufficient evidence in favour of one of the targets). Both of these types of errors may be exacerbated under time pressure (i.e. the instruction to respond as quickly as possible).

There is considerable evidence for the low prevalence effect outside of the laboratory setting (Horowitz, 2017), in clinical (Berlin, 1994; Evans et al., 2013; Gandomkar & Mello-Thoms, 2019), security (Fishel et al., 2015; Wolfe et al., 2013) and driving (Beanland et al., 2014) contexts (although see Clark et al., 2012; Hättenschwiler et al., 2019). In particular, Evans et al., (2013) tested expert mammographers in a real-world clinical setting during breast cancer screening checks and found that indeed, experts missed a much higher number of tumours in low prevalence conditions compared to high prevalence conditions. Similarly, newly trained Transportation Security Administration (TSA) officers were found to miss threat items of low prevalence during search for potentially dangerous objects in simulated airport security screening (Wolfe et al., 2013). Beanland et al. (2014) investigated the low prevalence effect in a driving context using a driving simulation test of visual search with target vehicles being either motorcycles or buses. Results indicated that drivers detected high-prevalence vehicles faster than low prevalence vehicles. Across all of these contexts, it appears that continuous performance assessment and training of professional visual search operators can improve their visual search efficiency for low-prevalence targets (Biggs & Mitroff, 2014; Biggs et al., 2013, 2018; Buser et al., 2020; Meuter & Lacherez, 2016; Mitroff et al., 2018; Nakashima et al., 2013; Spain et al., 2017).

The low prevalence effect has generally been explored during multiple target search (Kunar et al., 2017; Wolfe et al., 2007). In some instance of multiple target search, participants are presented with a visual display containing multiple targets which they have to identify (for example, Biggs et al., 2014). In other cases of multiple target search, participants have to maintain templates of multiple targets in their memory and only one of these targets will be present in a given search display (for example, Meener et al., 2010). Therefore, low prevalence search has been explored both in contexts where participants are searching for multiple targets in a given display and in contexts where they are searching for an instance of multiple possible targets held in memory. In visual search tasks, it has generally been observed that as the number of targets to be searched for increases, the less efficient the search becomes. This relative decrease in performance is referred to as a multiple-target cost (Ort & Olivers, 2020). For instance, Mestry et al. (2017) found that when searching for two faces simultaneously, as opposed to only one face, there are limitations in both capacity and guidance of visual search. Similarly, Menneer et al. (2009) compared the effectiveness of simultaneously searching for two target categories using X-ray images versus conducting two separate searches, one for each category. They found that when searching for both categories at the same time there was a cost in accuracy, but the extent of this cost varied depending on the characteristics of the targets. In particular, the performance of dual-target search was influenced by the representations of the targets required for the search. If the combined representations had conflicting values in the most important feature dimensions, there was a decline in performance. However, when the target representations shared features, the search was guided by common values, and hence, the cost for searching for two categories was reduced.

Godwin et al. (2010) investigated the low prevalence effect in a dual-target visual search task and found evidence supporting both the low prevalence effect (decreased target detection performance as target prevalence decreased) and the dual-target cost (simultaneously searching for two, rather than one, targets impairs search performance). When investigating the interaction of these two, results indicated that the efficiency of target detection in dual-target search was further limited by the prevalence effect, suggesting that when searching for two targets, the dual-target cost can be increased when these targets are of different prevalence.

The multiple-target cost observed when searching for multiple targets (Barrett & Zobay, 2014; Menneer et al., 2010; Mestry et al., 2017) can be explained using the theoretical account of attentional template prioritisation (Bundesen et al., 2005; Carlisle et al., 2011). Attentional templates are mental representations used to aid detection of task-relevant sensory inputs (Chelazzi et al., 1993, 1998). During multiple target search, mental representations of different targets are in competition, resulting in prioritisation of the search for some targets over others especially in cases where different targets have different levels of importance (Bays & Husain, 2008; Ma et al., 2014; Ort & Olivers, 2020; Williams et al., 2019). For example, Gruber et al. (2016) investigated how attentional templates for multiple target features versus single-target features are prioritised during target selection in visual search. The results suggested that attentional prioritisation for multiple-feature templates was less effective than that of single-feature templates of targets, both when target features remained constant and hence, could be represented in long-term memory, and when they changed across trials and therefore, had to be held in working memory. The authors therefore suggest that when observers are visually searching for multiple target representations, competition of target templates decreases effectiveness of visual search.

In a recent review by Huynh et al. (2021), it is argued that mental representations of different targets are weighed during visual search and prioritised according to their relevance to the task and goal-directed intensions of observers. It is possible that the weighting of attentional templates follows the principles of probability matching, also known as matching law or the principle of maximum likelihood (Herrnstein, 1961). Probability matching during visual search refers to a decision-making strategy whereby individuals distribute their attention or responses across different visual targets in proportion to the (perceived) probabilities of encountering those targets (Eriksen & Yeh, 1985; Jonides, 1980). Therefore, when weighing mental templates of multiple targets to decide which ones to prioritise during visual search, observers are likely to consider their perceived probability of encountering each item.

Given the important implications of the low prevalence effect in real-life contexts, vision researchers have recently started to investigate ways to overcome this effect. For example, as stated above, Fleck and Mitroff (2007) were able to ameliorate the low prevalence effect by providing participants the opportunity to correct their last response. Alternatively, Kunar et al. (2021) compared visual search performance of participants in ‘double reading’ procedures (where the visual search task was performed by two observers) versus ‘single reading’ procedures (where only one observer would perform the task) in a laboratory mammogram task. Asking participants to perform the task in pairs under the ‘double reading’ procedure led to a significant decrease in miss error rates compared to the ‘single reading’ condition. Finally, Menneer et al. (2007) found that a divided effort strategy, where different observers search for different target types, can significantly decrease miss error rates in low prevalence conditions compared to asking the same observer to search for all the different target types.

During visual search, endogenous (i.e. top-down cognitive influences) and exogenous (i.e. bottom-up perceptual influences) factors can ‘guide’ attention to different targets in the visual field (Wolfe et al., 1989). An important source of guidance during visual search, other than the prevalence of items, is the value associated with them (Anderson & Yantis, 2012; Anderson et al., 2011b) with its role being acknowledge by the latest Guided Search model 6.0 of Wolfe (2021). Offering a reward for detecting certain targets in visual search can direct more attention to them, improving visual search performance for those targets compared to the unrewarded or punished targets (Gong et al., 2016; Kiss et al., 2009; Krebs et al., 2010; Serences, 2008). This beneficial effect of reward has been observed when simple visual features (Anderson & Yantis, 2012; Anderson et al., 2011a, 2011b; Hickey et al., 2011; Laurent et al., 2015; Theeuwes & Belopolsky, 2012), locations (Chelazzi et al., 2014; Hickey et al., 2014) or complex objects (Hickey & Peelen, 2015; Hickey et al., 2015) are rewarded.

A few studies have investigated the influence of reward on prevalence effects and whether the detectability of a rare target can be enhanced by increasing the reward associated with it (Clark & Gilchrist, 2018; Navalpakkam et al., 2009; Won & Leber, 2016). For instance, Navalpakkam et al. (2009) investigated whether changing the reward outcomes in a simple visual search task with identical stimuli can improve detection rates. They found that increasing the reward offered for correct target detection can restore detection performance for rare targets, suggesting that reward schemes might be useful to improve detection rates in real-life tasks (however, see contradictory findings by Won and Leber (2016) and Clark and Gilchrist (2018). Similarly, Navalpakkam et al. (2010) compared the impact of value and prevalence on visual search in a complex perceptual environment. They found that observers were influenced by both the value and prevalence of targets in a manner consistent with the ideal (Bayesian) combination of these cues (i.e. participants combined both factors to maximise the expected reward within each trial).

In the majority of these past investigations where reward was used to ameliorate the prevalence effect, single-target searches were typically used. When trying to find ways to ameliorate the prevalence effect, it is important to use visual search paradigms where participants have to search for *multiple* targets as this more closely resembles the real-life contexts in which the low prevalence effect is likely to occur. In such situations, observers have to hold in memory and simultaneously search for multiple targets, each with a different degree of prevalence. For example, during airport security screening, TSA agents have to search for liquid bottles, which have a relatively high prevalence, while also searching for offensive weapons, which have a much lower prevalence. To our knowledge, only a limited number of studies have investigated the interaction of prevalence and reward during multiple target search, which is the aim of the current study.

In real-life contexts, it is often the case that different items also have different levels of importance associated with them (e.g. detecting a knife or gun in airport security screening is of much higher importance than detecting a liquid bottle). As a result, investigations of the interaction between prevalence and reward value should involve an *unequal reward pattern* such that prevalence of different targets is inversely related to the value associated with them. Wolfe et al. (2018) conducted a study looking at the impact of unequal value on the low prevalence effect using a hybrid foraging task with multiple instances of multiple targets. Interestingly, in a condition where both value and prevalence were inversely related, participants showed a preference towards collecting the most highly valued items, irrespective of their prevalence. This suggests that prioritisation of multiple targets in visual search may be malleable through reward manipulation. However, Wolfe et al. (2018) used a hybrid foraging task and not a MTS task. Hybrid foraging tasks have valuable differences with purer visual search tasks regarding both cognitive resources required (e.g. memory demands) and methodological characteristics (e.g. overall search times available; Gilchrist & Harvey, 2000; Gilchrist et al., 2001). For example, in the paradigm used of Wolfe et al. (2018) participants had no time limit during their search as they could choose when to move to the next item display after finishing their search in the previous one. As such, we were interested to explore the impact of reward on prevalence in a purer visual search task with multiple targets to be held in memory, where participants have a specific time limit for responding. These conditions resemble more closely some applied, real-life settings where participants are required to visually search for low prevalence targets under a tight time limit (e.g. during airport security screenings). Whether uneven rewards are enough to improve detection of low prevalence targets under tight time constraints, remains an open question.

In the current series of experiments, we investigated whether reward can be used to ameliorate the low prevalence effect in a Multiple Target Search (MTS) task where participants had to hold templates of multiple possible targets in their memory and search through displays in which only one of those targets, if any, would be present. The three different targets that participants were searching for had a different level of ‘priority’ (i.e. prevalence and/or reward) associated with them. Note that in the current paradigm due to the presence of multiple possible targets, we use the term prevalence to refer to the rate of a particular target appearing in the target-present trials (i.e. the percentage of trials containing a particular target). It is worth noting that this is similar to past investigations of visual search with multiple targets like Wolfe et al. (2018) who use the term ‘prevalence’ to refer to the percentage of trials containing a specific target. However, other authors have used the term prevalence to refer to the overall target probability (i.e. the percentage of trials containing a target, regardless of which target), and the term ‘frequency’ to denote a particular target item’s rate of appearance (i.e. the percentage of trials containing that specific target; e.g. Mitroff & Biggs, 2014; Wolfe et al., 2005).

As a first step in the current series of experiments, we replicated the basic low prevalence effect (Experiment 1) and reward effect (Experiment 2) in the current MTS paradigm. Subsequently, the interaction of prevalence and reward effect was explored (Experiments 3 and 4), testing whether and to what extent the low prevalence effect can be diminished, eliminated, or even reversed, through the manipulation of reward, in a task where participants are under a time constraint to respond. Experiments 3 and 4 provided further support for the robustness of the prevalence effect, although unequal rewards did diminish the low prevalence effect to some extent. Overall, this series of experiments showed that: (1) during MTS participants are able to prioritise search for mental representations of some targets over others based on their assigned priority (i.e. prevalence and/or reward), supporting past literature on relative prevalence (Godwin et al., 2010; Hout et al., 2015; Papesh et al., 2021; Walenchok et al., 2020) and reward (Gong et al., 2016; Kiss et al., 2009; Krebs et al., 2010; Serences, 2008) manipulations; (2) using the current magnitude of reward, prevalence has a stronger impact on MTS than reward value, but this impact can be diminished by an unequal reward pattern with high rewards assigned to lower prevalence targets.

## General method

### Transparency and openness

We report how we determined our sample size, all data exclusions, all manipulations, and all measures in each experiment, in accordance with the Transparency and Openness Promotion (TOP) Guidelines (Nosek, 2015). Each of the experiments was pre-registered on the Open Science Framework. Pre-registration information, the code for running the experiments, data and analysis scripts as well as stimuli used can be found on the OSF (Experiment 1: https://osf.io/cbueg/; Experiment 2: https://osf.io/gnjbx/; Experiment 3: https://osf.io/hrftb/; Experiment 4: https://osf.io/a3729/). All experiments reported here were granted ethical approval from the School of Psychological Science Research Ethics Committee at the University of Bristol. All experiments were conducted according to the revised Declaration of Helsinki (2013).

### Participants

Participants were recruited through the Prolific platform for online participant recruitment (https://www.prolific.co) over the period of mid to late 2021. They were paid £4 each for their participation (for Experiments 2–4 which had a reward component, participants were also accumulating points for entries to a £25 lottery). Previous lab-based studies involved sample sizes of around 18 participants (Clark & Gilchrist, 2018; Wolfe et al., 2007). As we conducted a series of online studies, we expected the data to be noisier, so we doubled this number and collected data from 36 participants for each experiment. In order to ensure that this sample size gave us enough power for the current MTS task, we performed a power calculation in R using the SIMR package suitable for an LME design (Green & Macleod, 2016). With an effect size of priority of 0.25 (derived from Experiment 1), a sample of 36 participants gives us at least 99% power of detecting a similar effect at an alpha of 0.05. Inclusion criteria for participation in the study included self-reported normal or corrected-to-normal vision and age between 16 and 35 years.

### Materials

**Fig. 1:**
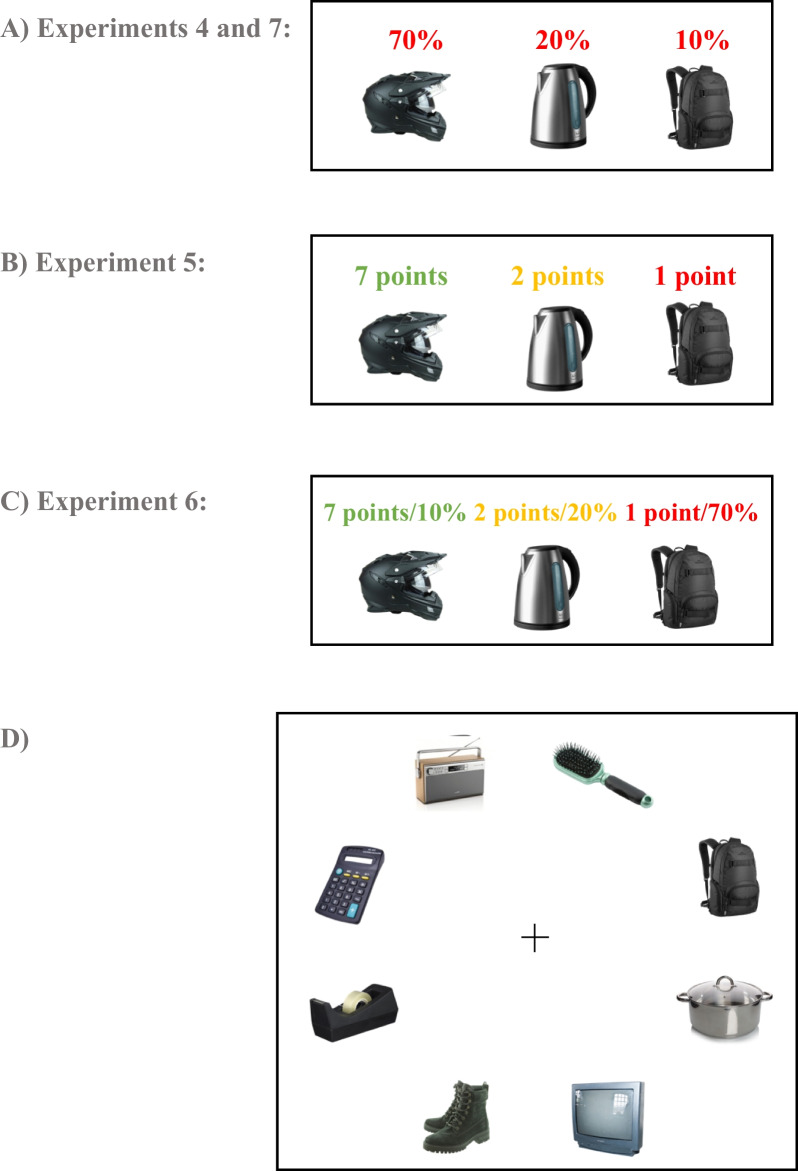
Panels **A**–**C**: A visual example of the instructions given to the participants in Experiments 1 to 4, regarding which three targets they would have to search for in the display together with their associated probabilities of appearing and/or reward associated with each. Panel **D**: An example of the stimuli displays during an experimental trial. After the display of 8 items, participants had to give a target-present or target-absent response as quickly as possible

A MTS task was programmed on Psychopy Builder (https://www.psychopy.org; (Peirce et al., 2019) and was run online on Pavlovia (https://pavlovia.org) on participants’ personal computers. On a typical 13″ laptop monitor with a screen resolution 1024 × 768 and viewing distance of 55 cm, one pixel corresponded to 0.02° of visual angle. Stimuli size is given based on this resolution and viewing distance, while size in PsychoPy height units is also reported. Eleven items were chosen that included portable items that one can find at home (i.e. a pair of shoes, a cup, a backpack, a TV, a tape, a kettle, a helmet, a radio, a calculator, a cooking pot and a hair brush). The items’ bounding box was square with sides of 1.57° of visual angle (0.1 height units). The same 11 items were viewed by all participants. For any one participant, three of these items were randomly chosen as targets of different prevalence and the remaining 8 items were distractors. On a given trial, there were 8 items presented on the display (see Fig. 1, Panel D for an example); in target-present trials, these items included 1 target and 7 randomly chosen distractors, whereas in target-absent trials, these items included all 8 distractors. The centres of the 8 items were evenly placed along the circumference of a circle with radius 6.28° of visual angle (0.4 height units), such that all items were placed at an equal distance from the centre (i.e. centre of the screen was the centre of the circle of stimuli).

### Procedure

Participants completed one testing session online, which lasted around 30 min. Participants had to remember three different targets throughout the experiment, and each of these targets was associated with a different level of prevalence and/or reward.^1^ These targets were randomly assigned to every participant but remained the same for the whole duration of the experiment. Participants were presented with these three targets at the beginning of every block as a reminder. On every trial, participants were presented with a central fixation cross of size 0.47° of visual angle (0.03 height units) for 1.5 s before the stimulus of the 8 different items was presented. The display with the 8 items remained on the screen until participants gave a response. Participants had to remember all three possible targets and detect if any one of the three targets (high, middle and low priority) was present or not and give a target-present (‘A’ key) or target-absent (‘L’ key) response as quickly as possible. Only one of these targets was present in each target-present trial. In each experiment, half the trials were target-present trials and half were target-absent trials (exact number of practice and experimental trials is given in the method section of each experiment).

In the current study, prevalence refers to the rate of a particular target appearing in the target-present trials (i.e. the percentage of trials containing a particular target). It is also worth clarifying that in the current MTS task, participants are searching for multiple *potential* targets as not all possible targets are visible in a given display (unlike similar foraging tasks; e.g. Wolfe et al., 2018). In order to ensure that participants were actually detecting a target and were not just able to guess, in Experiment 1, if participants gave a target-present response, they were presented with a second display of consecutive numbers from 1 to 8, of size 1.41° of visual angle (0.09 height units). Each one of these numbers was centred on the location where each item had previously been presented. Participants had to respond with the equivalent number key press to state in which particular location they detected the target. If they gave a target-absent response, then they would proceed straight to the next trial. In Experiments 2–4, this target localisation screen was presented randomly 5 times in each block. In Experiments 2–4, where a reward component was included, participants received feedback about their responses (more details to be given in the method section of each Experiment). The order of trial types was randomised within each block for every participant. Short breaks were allowed between blocks.

### Analysis plan

Linear Mixed Effects models (LMEs Baayen et al., 2008; Barr et al., 2013) and mixed-effect logistic regression models were used to analyse response time and accuracy data, respectively, using the lme4 package (Bates et al., 2015) for the R computing environment (R Development Core Team, 2015). Given the variations in subjects and stimuli in the current series of experiments, LMEs have been chosen as a more appropriate analysis method than traditional ANOVAs used in similar past investigations (e.g. Hout et al., 2015; Wolfe et al., 2007; Wolfe et al., 2017). For all four experiments, at the initial stage of analysis, we fit four different models with different structures and compared their AIC weight values in order to select the “best” (in terms of the trade-off between goodness of fit and parameter count) model structure for statistical inference. Table 1 outlines the different models which were explored, for the analysis of both response time and accuracy along with their AIC weights (Wagenmakers & Farrell, 2004) for Experiments 1–4. Model 1 was the null model which included only a random-intercept for participants and identity, without priority (prevalence and/or reward) being considered. Model 2 included priority (prevalence and/or reward) as a fixed effect and a random-intercept for participants and identity. Model 3 included random-intercepts for participants and identity as well as priority (prevalence and/or reward) as fixed effect and as a random slope for participants. Model 4 was the maximal model that included random-intercept for both participants and identity as well as priority (prevalence and/or reward) as a fixed effect and as a random slope by both participants and identity. Results from the model with the highest AIC weight value are reported in the results section of each experiment (Burnham & Anderson, 2004). All inferences made from the findings of each experiment are based on the winning model.^2^

**Table 1:** AIC weight values for all models explored in Experiments 1–4

Experiments	Models			
	1	2	3	4
	(null)	(+ random intercept)	(+ random slope)	(maximal)
*Response time (LME analysis)*				
1	0	0	**0.91**	0.09
2	0	0	**1**	0
3	0	0.16	**0.77**	0.08
4	0	**0.57**	0.42	0.01
*Accuracy (Mixed effect logistic regression)*				
1	0	0.07	**0.99**	0
2	0	0.06	**0.98**	0
3	0	0	**0.98**	0.02
4	0	0	**0.96**	0.04

The model which best captures the data in each experiment and variable is the model with the highest AIC weight and is highlighted in bold

Given the use of natural images and the random assignment of targets/distractors to different participants, it is important to account for any variance in performance induced by variations in the detectability of the different objects. Therefore, data were not aggregated across trials.^3^ For both response time and accuracy measures, data were analysed on a trial level. In Experiments 1, 2 and 4, priority (i.e. prevalence or reward) was entered as a predictor factor with low priority condition as the reference category. For Experiments 3, where both prevalence and reward were manipulated, we refer to the predictor as the ‘status’ of targets. Target status was entered as a factor with low prevalence/high reward condition as the reference category. In both LME (i.e. response time data) and multiple logistic regression (i.e. accuracy data) analyses, we use sliding differences (i.e. repeated) contrasts instead of default treatment contrasts in order to explore differences across the three different levels of priority. Using sliding differences contrasts, the analyses explore differences between the reference category (i.e. low priority) and the second (i.e. middle priority) category as well as differences between the second category (i.e. middle priority) and third category (i.e. high priority). For each measure, we report fixed effect estimates for each prevalence level from the chosen model. The same analysis plan and model structure was used for all four experiments.

For the response time data, restricted likelihood and Nelder–Mead optimisation was used and response time of participants in correct target-present trials was entered as the dependent variable. To deal with the skewness of the response time data and following results of the Box-Cox (1964) test, a reciprocal transformation was applied (only for the purpose of statistical analyses; descriptive statistics and figures are based on the untransformed data). Accuracy of the present/absent response in target-present trials was analysed using a mixed-effects logistic regression and proportion of miss errors of participants in correct target-present trials was entered as the dependent variable. BOBYQA optimisation was used. Correct responses were coded with 0, and incorrect responses were coded with 1.

## Experiment 1

Experiment 1 aimed to replicate the basic low prevalence effect in the current MTS task. Priority was manipulated in terms of prevalence, with participants having to hold mental templates of three different targets with different degrees of prevalence: high (70% occurrence when a target was present), medium (20% occurrence) and low (10% occurrence) prevalence. Using three levels of prevalence allowed us to assess to what extent participants were able to prioritise targets in either a graded manner or an all-or-none manner with some (or even all) target templates prioritised to the same degree. It was expected that as prevalence of targets increased, participants response time for correct target-present responses would decrease and fewer miss errors (i.e. better accuracy) would be observed.

### Method

#### Participants

Thirty-six participants (14 female) took part in the experiment, with age (M ± SD), 24.1 ± 3.9 years.

#### Design

The within-subjects priority factor of prevalence was manipulated across three levels (Low: 10%, Middle: 20% and High: 70%).

#### Procedure

Participants completed 20 practice trials to familiarise themselves with the computer task followed by 400 experimental trials. Half the trials were target-present trials, and half were target-absent trials. These 400 experimental trials were divided into 5 blocks of 80 trials each (i.e. 40 target-present and 40 target-absent trials in every block). Each block contained an equal number of trial types: 28 trials (70%) where a high prevalence target was presented, 8 trials (20%) where a medium prevalence target was presented, 4 trials (10%) where a low prevalence target was presented and 40 trials where 8 distractors and no target were presented. At the beginning of every block, participants were presented with the templates of the three targets they would have to search for in the visual display, and the associated probabilities of each of the three targets appearing (i.e. low: 10%, middle 20%, high: 70%; see Fig. 1, Panel A).

### Results

Trials in which participants had a response time less than 200 ms or more than 6 s were excluded from the analysis. Based on this exclusion criterion, 1.8% of trials were excluded. Participants, who had more than 10% of their trials excluded, were removed from the analysis. Based on this exclusion criterion, 2 participants were excluded from the total of 36 participants. Because the pre-registration for this Experiment did not include any exclusion criteria, data were also analysed *before* exclusion and a qualitatively similar pattern of results to that reported here was obtained. In the subsequent experiments, the same exclusion criteria were registered and applied. To ensure comprehensible comparison between findings of all three experiments, reported results of Experiment 1 include data analysed *after* application of the exclusion criteria.

Out of the four different models explored in the analysis, Model 3 was found to capture the data from Experiment 1 best, for both response time and accuracy, as seen in Table 1. This model included random intercepts for participants and target identity as well as prevalence as fixed effect and as a random slope for participants. Results from this model are reported for each variable.

#### Response time analysis

**Fig. 2:**
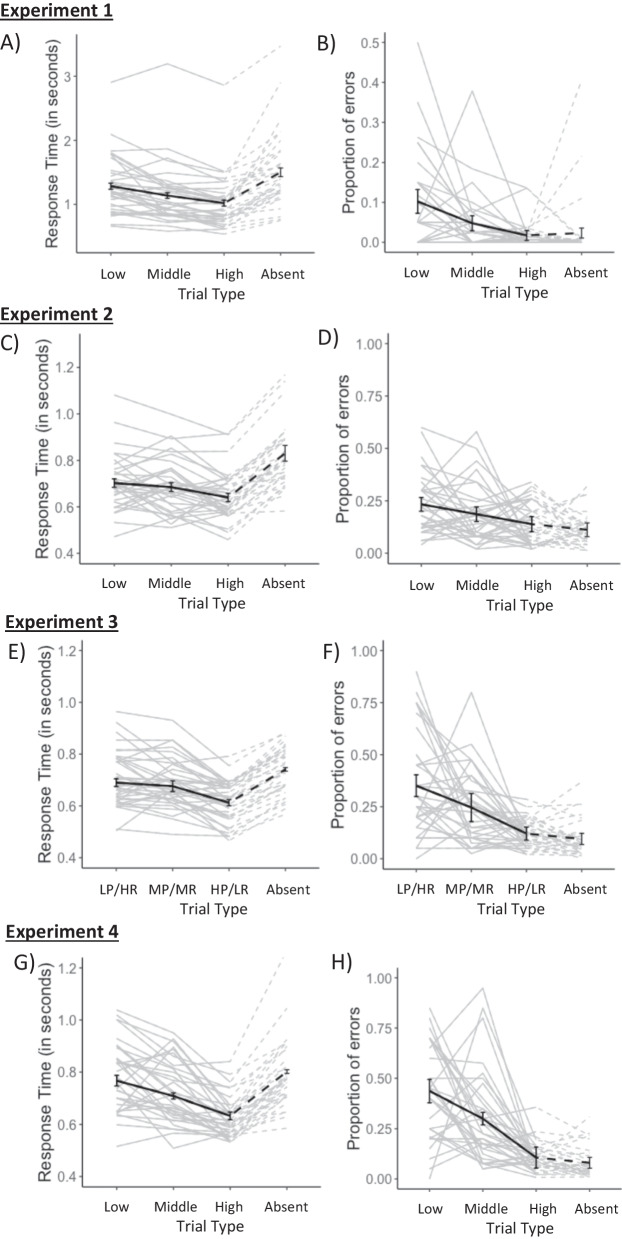
Lefthand Panels (i.e. **A**, **C**, **E**, **G**): Mean Response Time (in seconds) for all participants in target-present (i.e. low, middle and high prevalence and/or reward) and target-absent trials in Experiments 1–4, respectively. Righthand Panels (i.e. **B**, **D**, **F**, **H**): Proportion of errors, for all participants in Experiments 1–4, respectively. In target-present trials (i.e. low, middle and high prevalence and/or reward), these refer to miss errors whereas in target-absent trials these refer to false alarms. In all panels, black lines indicate mean measures across all participants, while grey lines indicate individual data for each participant. Error bars represent 95% within-subject confidence intervals following Morey (2008)

Figure 2 (Panel A) indicates response time for hit (i.e. correct target-present trials) across all three prevalence levels or correct rejection (i.e. correct target-absent trials) across all three prevalence levels. As prevalence increased in target present trials, participants were significantly quicker at correctly identifying the target. The slowest response time was observed in the low prevalence condition (*b* = 1.08, SE = 0.05, *t* = 20.09, *p* < 0.001), an intermediate response time was observed in middle prevalence condition (*b* = 0.11, SE = 0.02, *t* = 4.56, *p* < 0.001), and the fastest response time was observed in high prevalence condition (*b* = 0.14, SE = 0.02, *t* = 7.43, *p* < 0.001).

#### Accuracy analysis

Figure 2 (Panel B) indicates participants proportion of errors across all trial types. In target-present trials, these refer to miss errors, while in target-absent trials, these refer to false alarms. It can be seen that as prevalence increases, participants’ accuracy in target detection is improved as their miss errors in target-present trials decrease. Prevalence was found to have a significant effect on participant’s accuracy, whereby the lowest accuracy was observed in the low prevalence condition (log odds = − 3.67, SE = 0.26, *z* = − 14.23, *p* < 0.001), an intermediate accuracy was observed in the middle prevalence condition (log odds = − 1.01, SE = 0.33, *z* = − 3.08, *p* = 0.002) and the highest accuracy was observed in the high prevalence condition (log odds = − 1.12, SE = 0.29, *z* = − 3.83, *p* < 0.001). Overall, the average proportion of miss errors across participants in target-present trials decreased as prevalence increased (Fig. 2, Panel B).

### Discussion

Taken together, the results of Experiment 1 support the existence of the low prevalence effect in the current MTS task, demonstrating that participants had slower response times and a higher proportion of misses when the target had a low compared to high prevalence. This is in line with the previous literature, suggesting that observers are quicker and more accurate in target detection, for high versus low prevalence targets (Clark & Gilchrist, 2018; Wolfe et al., 2005, 2007). Current results even extend past literature findings as they indicate that when searching for *multiple* targets, visual search performance of participants increases as prevalence increases in a graded manner, while similar past literature findings primarily involved the search of maximum two targets (Hout et al., 2015). This means that participants are capable of searching for targets in an unequal and graded manner, prioritising search for some targets versus others depending on their associated levels of importance.

## Experiment 2

Having established the low prevalence effect in the current MTS task, the purpose of Experiment 2 was to investigate the influence of reward on target detection. In particular, this experiment aimed to replicate the basic effect of reward on target detection (Kiss et al., 2009; Krebs et al., 2010) in the current visual search task with multiple targets. Priority was manipulated in terms of the amount of reward received upon both accurate and quick target detection. Participants had to hold representations in memory of three different targets with different degrees of reward (i.e. high, medium and low reward). This allowed us to test whether participants were able to prioritise these targets in a graded manner in accordance with their reward level or whether prioritisation followed an all-or-nothing pattern with some (or even all) target templates prioritised to the same degree. It was expected that as reward associated with targets increased, participants response time for correct target-present responses would decrease and fewer miss errors (i.e. better accuracy) would be observed.

### Method

#### Participants

Thirty-six participants (21 female) took part in the experiment, with age (M ± SD), 26.3 ± 4.3 years.

#### Design

The within subject priority factor of reward was manipulated across three levels (Low: 1 point, Middle: 2 points and High: 7 points).

#### Procedure

Participants completed 20 practice trials to familiarise themselves with the task followed by 300 experimental trials. A total of 150 of those were target-present trials, and 150 of those were target-absent trials. Out of the 150 target-present trials, 50 contained the high reward target, 50 the middle reward target and 50 the low reward target (i.e. prevalence remained constant).^4^ These 300 experimental trials were divided into 5 blocks of 60 trials each with an equal number of each trial type. The order of trial types was randomised within each block for each participant. There were opportunities for breaks between blocks.

In both target-present trials and target-absent trials, if participants gave a correct and quick response, they would receive a reward, in points. These points were then translated to lottery tickets (1 point = 1 lottery ticket). At the end of every block participants were presented with the total number of tickets, they had collected so far. Participants were told that three lottery tickets from all participants would be chosen at random, with the constraint that the tickets must belong to three different participants, and the three winners would receive £25 extra for their participation in the experiment. Therefore, the more tickets participants collected the higher their chance of winning. The response time benchmark for assigning reward was less than or equal to 1000 ms (this criterion was set to fall just below the average median response time of all participants in Experiment 1, which was 1100 ms). This element of time pressure for reward allocation was inserted to more closely resemble real-life settings where participants are required to visually search for low prevalence targets under a time limit (e.g. during airport security screening), as well as to prevent participants from purposely taking an unreasonably long amount of time to give a response in an effort to maximise their accuracy and gain the reward. The time requirement is kept for Experiments 3 and 4 as well.

At the beginning of every block, participants were presented with the images of the three targets they had to search for in the visual display, and the associated levels of reward in terms of points (see Fig. 1, Panel B for an example). The colour of the text denoting the number of points for each target varied such that ‘7 points’ was written in green colour, ‘2 points’ was written in orange colour and ‘1 point’ was written in red colour. In target-present trials, if participants received a reward they were presented with written feedback about the number of points they received (depending on which target was shown). The text colour used in the visual feedback was the same as the associated level of reward. In target-absent trials, if participants gave a correct and quick enough response, they also received a reward that was equally likely to be low (1 point), medium (2 points) or high (7 points). This was done in order to motivate participants to pay attention to target-absent trials as well and discourage them from continuously giving target-present responses. In this case, the text colour used in the visual feedback was black. In both target-present and target-absent trials, along with the visual notification of their reward if a correct and quick reply was given, participants heard a ‘coin drop’ sound, notifying them that they have received points.

If participants gave an incorrect response in both target-present and target-absent trials, they were presented with a feedback screen notifying them that no reward had been received. In this case instead of the ‘coin drop’ sound participants heard a low frequency sine wave sound, notifying them about their incorrect response. If participants gave a correct but too slow response (i.e. greater than 1 s), they received no reward and they were presented with an written feedback that they were too slow and no feedback sound was played.

Visual and auditory feedback was available for participants for 1.5 s. Visual text feedback was located at the centre of the screen and was written in Times New Roman font with a size of 1.1° of visual angle (which corresponds to 0.07 height units). The actual volume of the sound participants heard, depended on the volume of their individual computers. However, the scale factor for sound feedback was set at 1, which means that the sound would play at the exact volume set on participants’ computer. PsychoPy PTB audio library was chosen, and audio latency priority was set at 4. Sounds for audio feedback were retrieved from an online sound-effect library (https://freesound.org/people/Bertrof/sounds/351565/).

### Results

From Experiment 2 onwards, the presence of a temporal deadline for reward allocation speeded up the responses of participants and consequently increased their overall error rates. Therefore, an additional exclusion criterion regarding accuracy had to be applied to ensure that analysis did not include trials in which participants sacrificed accuracy too much in order to meet the temporal deadline. The same exclusion criteria as Experiment 1 were applied regarding response time, with the following exception. Given the presence of the temporal deadline for reward assignment (i.e. 1,000ms) in this Experiment, participants’ responses were speeded up compared to Experiment 1; therefore, it was more appropriate to decrease the response time upper limit for exclusion which was now set to 4,000ms. 0.1% trials were excluded based on this criterion as too fast or too slow. No participants had more than 10% of their trials excluded. An additional exclusion criterion to Experiment 1 was applied regarding accuracy of participants (this criterion is included in the pre-registration for all experiments in this series with a reward element, Experiments 2, 3 and 4). Based on participants’ accuracy distribution, those with a hit rate below 2 SDs of the mean or a false alarm rate above 2 SDs of the mean were excluded from further analysis. Based on this exclusion criterion, three participants were excluded from the analysis (one participant had a hit rate below 2SDs of the mean and two participants had a false alarm rate above 2SDs of the mean).

Out of the four different models explored in the analysis, Model 3 again capture the data from Experiment 2 best, regarding both response time and accuracy measures, as seen in Table 1. This model included random intercepts for participants and identity as well as reward as fixed effect and as a random slope for participants. Results from this model are reported for each variable.

#### Response time analysis

Figure 2 (Panel C) shows the response time of participants in trials where they gave a correct hit or correct rejection. A main effect of reward on participants’ response time was found, whereby the slowest response time was observed in low reward condition (*b* = 1.58, SE = 0.05, *t* = 34.33,* p* < 0.001), an intermediate response time was observed in middle reward condition, although this contrast was not significant (*b* = 0.04, SE = 0.02, *t* = 1.64, *p* = 0.120). The fastest response time was observed in high reward condition (*b* = 0.08, SE = 0.03, *t* = 2.95, *p* < 0.001). This evidence suggests that as reward levels increased, response time decreased (Fig. 2, Panel C). The slowest average response time was observed in target-absent trials, in line with previous literature (Wolfe et al., 2005, 2007).

#### Accuracy analysis

Figure 2 (Panel D) shows participants proportion of errors across all trial types. As reward increases, participants’ accuracy in target detection is improved as their misses decrease. Findings indicated that reward had a significant effect on participant’s accuracy, whereby the lowest accuracy was observed in the low reward condition (log odds = − 1.69, SE = 0.18, *z* = − 9.22, *p* < 0.001), an intermediate accuracy was observed in the middle reward condition (log odds = − 0.43, SE = 0.16, *z* = − 2.75, *p* = 0.006) and the highest accuracy was observed in the high reward condition (log odds = − 0.22, SE = 0.20, *z* = − 1.09, *p* < 0.001). As reward increased, participants’ likelihood of making an error decreased (Fig. 2, Panel D).

### Discussion

The results of Experiment 2 support the prediction that participants would have faster response times and fewer misses in identifying the target amongst distractors when this is associated with a higher versus lower reward. This is in line with the previous literature, suggesting that observers are quicker and more accurate in target detection as reward increases (Gong & Yang, 2016; Kiss et al., 2009; Krebs et al., 2010; Serences, 2008). The results of the current experiment support the claim that participants are able to prioritise visual search for some mental representations of targets versus others, in a graded manner according to the priority (i.e. reward) assigned to each.

## Experiment 3

Having established the effects of prevalence (Experiment 1) and reward (Experiment 2) on target detection in the current modified MTS task, the aim of Experiment 3 was to combine both prevalence and reward effects to explore whether detection efficiency of low prevalence targets can be improved through reward. A few studies have investigated whether reward can improve detection of low prevalence targets; however, evidence is not yet conclusive regarding the extent to which reward can overcome or moderate the low prevalence effect (Clark & Gilchrist, 2018; Navalpakkam et al., 2009; Won & Leber, 2016). To our knowledge, limited investigations have been conducted where unequal reward is used to mitigate the low prevalence effect (Wolfe et al., 2018). In the current experiment, reward was inversely related to the prevalence of a target such that as prevalence decreased reward increased. There were two distinct possible outcomes from the current study; a) *prevalence* would trump reward in which case reward would no longer influence performance at all and we would expect similar results to Experiment 1 or b) *reward* would modulate the low prevalence effect in that the low prevalence effect would get diminished, eliminated or even reversed. Consider the negative slope(s) relating response time and errors to prevalence. The question is: what happens with this slope in the case where reward is inversely related to prevalence? In the diminution scenario, reward would decrease the influence of prevalence on target detection but not to the extent that it would eliminate or even reverse its effect (negative slope). In the case of elimination, then the increase in reward for the low prevalence target would be exactly right to counteract its lower prevalence (approximately zero slope). Given the prevalence and reward values chosen in this experiment, this would suggest that participants are guided by expected value (the product of the probability of an event occurring and the magnitude of reward associated with it). In the scenario of complete reversal, then reward would trump prevalence (positive slope) and we would expect results similar to Experiment 2.

### Method

#### Participants

Thirty-six participants (18 female) took part in the experiment, with age (M ± SD), 23.6 ± 3.6 years.

#### Design

Target status (i.e. prevalence/reward combination) was manipulated within subjects, across three levels: low prevalence/high reward, medium prevalence/medium reward and high prevalence/low reward.

#### Procedure

Participants were informed about the associated prevalence and reward of each target at the beginning of the experiment and before the start of each block as a reminder, similar to Experiments 1 and 2 (see Fig. 1, Panel C for an example). Participants completed 20 practise trials followed by 400 experimental trials. Prevalence was manipulated in the same way as in Experiment 1 where out of 400 experimental trials, 200 were target-present and 200 were target absent. High prevalence targets were present on 70% of target-present trials (i.e. 140 trials), a medium prevalence target was present on 20% of target-present trials (i.e. 40 trials) and a low prevalence target was present on 10% of target-present trials (i.e. 20 trials). The same visual and auditory reward cues were used as in Experiment 2, but in this case high reward was assigned to the low prevalence target, medium reward to the medium prevalence target and low reward to the high prevalence target. In target-absent trials, if participants gave a correct and quick enough response, they also received a reward. However, due to the different number of trials in Experiment 3 versus Experiment 2, in this case the amount of points gained was *almost* equally likely to be low, medium or high (i.e. 65 trials would be rewarded with 1 point, 70 trials would be rewarded with 2 points and 65 trials would be rewarded with 7 points).

### Results

The same exclusion criteria to Experiment 2 was applied. 0.1% of the trials were excluded as too fast or too slow. No participants had more than 10% of their trials excluded. Based on participants’ accuracy distribution, those with a hit rate below 2 SDs of the mean and a false alarm rate above 2 SDs of the mean were excluded from the analysis. Based on this exclusion criterion, two participants were excluded from the analysis.

Out of the four different models explored in the analysis, Model 3 was found to best capture the data in Experiment 3, regarding both response time and accuracy measures, as seen in Table 1. This model included random intercepts for participants and identity as well as target status as fixed effect and as a random slope for participants. Results from this model are reported for each variable.

#### Response time analysis

Figure 2 (Panel E) indicates the response time of participants in trials where they gave a correct hit or correct rejection. As prevalence increased in target present trials, participants were significantly quicker at correctly detecting it, irrespective of the higher reward offered to low prevalence targets. A main effect of status on participants’ response time was found, whereby the slowest response time was observed in low prevalence/high reward condition (*b* = 1.62, SE = 0.04, *t* = 38.19, *p* < 0.001), an intermediate response time was observed in the medium prevalence/ medium reward condition (*b* = 0.07, SE = 0.03, *t* = 2.30, *p* = 0.029) and the fastest response time was observed in the high prevalence/low reward condition (*b* = 0.16, SE = 0.02, *t* = 7.78, *p* < 0.001). This evidence suggests that as prevalence levels increased, response time decreased (Fig. 2, Panel E) similar to the effect of prevalence observed in Experiment 1, irrespective of the reward given in the current experiment. This suggests that reward did not moderate the effect of prevalence on target detection.

#### Accuracy analysis

Figure 2 (Panel F) shows the proportion of errors across all trial types. It can be seen that as prevalence increases, participants’ errors in target detection decreases, irrespective of the higher reward associated with low prevalence targets. There was a main effect of target status on participants’ accuracy, whereby the lowest accuracy was observed in the low prevalence/high reward condition (log odds = − 1.39, SE = 0.15, *z* = − 9.32, *p* = 0.001), an intermediate accuracy was observed in the medium prevalence/medium reward condition (log odds = − 0.58, SE = 0.258, *z* = − 2.27, *p* = 0.024), and the highest accuracy was observed in the high prevalence/low reward condition (log odds = − 0.76, SE = 0.19, *z* = − 4.10, *p* < 0.001). This indicates that as prevalence increased, participants’ likelihood of making an error decreased; hence, accuracy in target detection was improved, regardless of the reward assigned (Fig. 2, Panel F).

### Discussion

Overall, both response time and accuracy measures in Experiment 3 suggest that with the current reward pattern in this modified MTS task, prevalence dominates reward. The results resemble closely those of Experiment 1: faster responding and fewer misses were observed as prevalence increased. However, response times and error rates for Experiment 1 are very different from those in Experiments 2 and 3 because from Experiment 2 onwards, there was a temporal deadline (if participants did not respond within 1000 ms, they did not receive the reward), whereas there was no explicit time pressure in Experiment 1. So in Experiments 2 and 3 participants respond more quickly and had significantly lower response times and higher overall error rates, compared to Experiment 1 (Fig. 2). Therefore, it was difficult to compare the data from Experiment 1 to the rest as participants approached the task fundamentally differently in Experiment 1 (even though they were instructed to respond as quickly as possible, they were not trying to be quite as fast as the participants in Experiments 2 and 3 who had to respond with a deadline to obtain reward and who also received feedback about their response speed).

Due to these differences between Experiments 1 and 3, the current results do not allow us to make conclusive inferences on the actual impact reward had on the prevalence effect. It is possible that prevalence is found to be stronger than reward. However, the results do not rule out the scenario that reward diminishes prevalence, at least to a degree (i.e. diminution scenario), such that it does not lead to complete elimination or even reversal of the prevalence effect. As a result, it is critical to assess the low prevalence effect in the presence of equal reward and the same time pressure that participants were under in Experiment 3. Such a manipulation makes for a cleaner test of the extent to which the low prevalence effect may be modulated by unequal reward.

## Experiment 4

We are specifically interested in the effect of *unequal reward* as a way of counteracting the effect of prevalence. In the current experiment, a *constant* reward will be associated with all targets. Experiment 4 is designed as an attempt to equate for the presence of a temporal deadline, the presence of a reward and feedback about response speed. In this way, we aim to make the conditions as similar as possible to Experiment 3, except for the unequal reward. Given that the reward associated with all targets is the same in the current experiment (irrespective of their prevalence), we expect to see simply the standard prevalence effect. If unequal reward (as in Experiment 3) influences the prevalence effect, compared to equal reward (i.e. Experiment 4), so that it can attenuate the low prevalence effect, then we would expect higher response time and miss errors (i.e. worse accuracy) in the current Experiment, particularly, in the low prevalence condition.

### Method

#### Participants

Thirty-six participants (24 female) took part in the experiment, with age (M ± SD), 23.5 ± 4.2 years.

#### Design

Target prevalence was manipulated within subjects across three levels (Low, Medium and High) as in Experiment 1. However, in this experiment a constant reward (i.e. number of points) was associated with all targets, unlike Experiment 1 in which no reward was used.

#### Procedure

Participants were informed about the associated prevalence of each target at the beginning of the experiment and before the start of each block as a reminder, as in previous experiments (see Fig. 1, Panel A for an example). Participants completed 20 practise trials followed by 400 experimental trials. Out of which 200 were target-present and 200 were target absent. The three different targets participants had to search for in the visual display were associated with three levels of prevalence as in Experiments 1 and 3, such that the high prevalence target was presented on 70% of the trials, the medium prevalence target was presented on 20% of the trials, and the low prevalence target was presented on 10% of the trials.

In the current experiment, a constant reward of 2 points was given to the participants upon quick (i.e. less than 1 s) and accurate responding in both target-present and target-absent trials, as opposed to Experiment 3, where a larger reward was associated with low prevalence targets and less reward was associated with high prevalence targets. Visual and auditory feedback was presented to the participants in the same way as in Experiment 2 and 3, but in this case, the text colour used in the visual feedback was always black as there was only one reward level.

### Results

The same exclusion criteria to Experiments 2 and 3 was applied.^5^ 0.2% of the trials were excluded as too fast or too slow. No participants had more than 10% of their trials excluded. Based on participants’ accuracy distribution, those with a hit rate below 2 SDs of the mean and false alarm rate above 2 SDs of the mean were excluded from analysis. Based on this exclusion criterion, three participants were excluded from the analysis (i.e. two participants had a hit rate below 2SDs of the mean and 1 participant had a false alarm rate above 2SDs of the mean).

Out of the four different models explored in the analysis, Model 2 was found to best capture the data regarding response time measure (Table 1). This model included prevalence as a fixed effect and a random intercept for participants and identity. Regarding accuracy measure, Model 3 was found to best capture the data (Table 1). This model included random intercepts for participants and identity as well as prevalence as fixed effect and as a random slope for participants. Results the winning model are reported for each variable, respectively.

#### Response time analysis

Figure 2 (Panel G) shows response time for participants in trials where they gave a correct hit or correct rejection. As prevalence increased, participants were significantly quicker at correctly detecting the target. Analysis indicated a main effect of prevalence on participants’ response time, whereby the slowest response time was observed in the low prevalence condition (*b* = 1.55, SE = 0.04, *t* = 36.89, *p* < 0.001), an intermediate response time was observed in medium prevalence condition (*b* = 0.09, SE = 0.02, *t* = 3.78, *p* < 0.001) and the fastest response time was observed in the high prevalence condition (*b* = 0.19, SE = 0.01, *t* = 13.18, *p* < 0.001). This evidence suggests that as prevalence levels increased, response time decreased (Fig. 2, Panel G) similar to the effect of prevalence observed in Experiments 1 and 3.

#### Accuracy analysis

Figure 2 (Panel H) shows participants proportion of errors across all trial types. As prevalence increases, participants’ accuracy in target detection increased as their miss rate in target-present trials decreases, indicating a main effect of target prevalence on accuracy, whereby the lowest accuracy was observed in the low prevalence condition (log odds = − 1.18, SE = 0.23, *z* = − 5.18, *p* < 0.001), an intermediate accuracy was observed in the medium prevalence condition (log odds = − 0.71, SE = 0.27, *z* = − 2.61, *p* = 0.009) and the highest accuracy was observed in the high prevalence condition (log odds = − 1.29, SE = 0.17, *z* = − 7.69, *p* < 0.001). This means that the average proportion of miss errors across participants in target-present trials decreased as prevalence increased (Fig. 2, Panel H).

Looking at the main findings of the Experiments 3 and 4, faster response times and fewer errors are observed in the low prevalence condition of Experiment 3 (Figs. 2, Panel E and Panel F, respectively) than in the low prevalence condition of Experiment 4 (Figs. 2, Panel G and Panel H, respectively), suggesting that equal (Experiment 4) and unequal (Experiment 3) reward had a different impact on the influence of prevalence on visual search. As a result of this observation, some further exploratory analysis was conducted.

#### Exploratory analysis

**Fig. 3:**
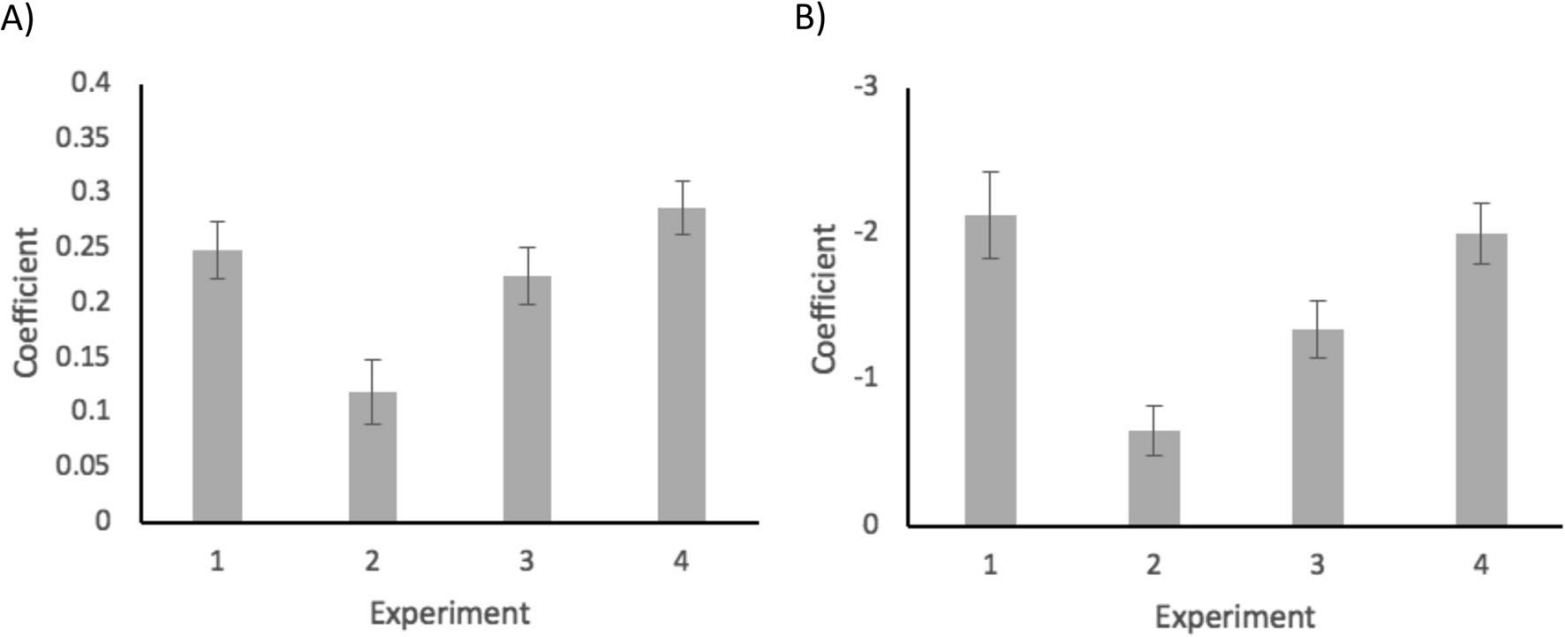
Panel **A** Coefficients represent beta values from LME analysis of response time. Panel **B** Coefficients represent log odds from multiple logistic regression analysis of proportion of miss errors (i.e. incorrect responses in target-present trials only). In both panels, regression coefficients of the high priority condition relative to the low priority condition (reference category) are plotted. In Experiments 1, 3 and 4, high priority refers to high prevalence condition and in Experiment 2, high priority refers to high reward condition. Error bars represent standard error

Further exploratory analysis was conducted regarding the effect of priority (prevalence in Experiments 1, 3 and 4; reward in Experiment 2) across all four experiments. The primary aim of this exploratory analysis was to compare the extent to which prevalence influences visual search performance across Experiments 3 and 4 in which reward was manipulated in an unequal (i.e. high reward associated with low prevalence targets and low reward associated with high prevalence targets) and equal (i.e. constant reward associated with low, middle and high prevalence targets) manner, respectively. Figure 3 illustrates the coefficients (i.e. beta values from LME analysis of response time in Panel A and log odds from multiple logistic regression analysis of proportion of miss errors in Panel B) of high priority conditions relative to low priority conditions (reference category) using standard treatment contrast coding. These coefficients show the effect of priority on response time and accuracy of participants between the *high* and *low* priority conditions. The smaller beta values of the LME analysis in Experiment 3 compared to Experiment 4 show a smaller effect of prevalence on participants’ response time when reward was manipulated unequally (Experiment 3) versus when reward was manipulated equally (Experiment 4). Similarly, the log odds of the multiple regression analysis on participants’ accuracy indicate the chance of making an error in each experiment, with smaller values suggesting a smaller probability of making an error. In particular, the smaller log odds of accuracy in Experiment 3 compared to Experiment 4 indicate a smaller effect of prevalence on participants accuracy when reward was manipulated unequally (Experiment 3) versus when reward was manipulated equally (Experiment 4). This highlights that offering a higher reward to low prevalence targets and a lower reward to high prevalence targets, decreased the influence of prevalence on target detection but not to the extent that it would eliminate or reverse the effect of prevalence.

Given the clear difference of the effect of prevalence between Experiment 3 and 4, a further exploratory analysis was performed exclusively on the data from these two experiments. In particular, LME and mixed-effect logistic regression analyses were run for response time and accuracy measures, respectively, with ‘experiment’ as a between-subjects factor where Experiment 3 was coded as 0 (reference category) and Experiment 4 was coded as 1. Prevalence was entered as a within-subjects factor with the low prevalence condition as the reference category. In the mixed-effect logistic regression, BOBYQA optimisation was used and correct responses were coded with 0 while incorrect responses were coded with 1. This analysis aimed to investigate a potential interaction between the within-subject factor of prevalence and the between-subject factor of ‘experiment’. Such an interaction would suggest that prevalence influences visual search performance of participants differently across experiments because of the different reward structure. Different models were explored for the analysis of both response time and accuracy and were assessed based on their AIC weights. The structure for each model was the same as the one already specified in the analysis plan section and displayed in Table 1. Model 3 investigated the effects of prevalence and experiment (as a between-subjects factor), as well as the interaction of these two variables, using random intercepts for participant and target identity and prevalence as a random slope for participants. Model 3 captured the data better than all other models for both response time and accuracy measures. Effect estimates for all levels of both prevalence and experiment variables from the winning model were used for the inferences made below. For both response time and accuracy measures, data were again analysed at the trial level.

#### Response time measures

The critical interaction between the within-subject factor of prevalence and the between-subject factor of experiment (*b* = − 0.12, SE = 0.05, *t* = − 2.22, *p* = 0.03) was significant: the difference between low and high prevalence was more pronounced in Experiment 4 compared to Experiment 3 (*b* = 0.08, SE = 0.03, *t* = 2.51, *p* = 0.015).

#### Accuracy measures

Findings suggested an important interaction between the within-subject factor of prevalence and the between-subject factor of experiment (log odds = 0.42, SE = 0.28, *z* = 1.49, *p* = 0.134), indicating that accuracy of participants significantly differed across the two experiments between the low and high prevalence conditions (log odds = − 0.61, SE = 0.26, *z* = − 2.33, *p* = 0.019).

In a final exploratory analysis, we investigated whether repetition effects were driving the benefit for the high prevalence targets. It is well established that participants in visual search tasks can often exhibit repetition effects, whereby participants respond faster and more accurately when a target stimulus is repeated on sequential trials (Hillstrom, 2000; Kristjánsson & Campana, 2009; Malgkovic & Nakayama, 1994). Trials with high prevalence targets are more likely to be repeated than medium or low prevalence trials. Therefore, we performed an LME analysis on participants’ response times in Experiment 4, on trials where the high prevalence target appeared after a different trial type (no repetition) or appeared for two, three or four consecutive trials. ‘Repetition’ was used as a predictor factor with four levels: zero, one, two and three; and the reciprocal response time of participants for correct trials only was entered as a dependent variable. The level of ‘zero repetitions’ was used as the reference category, while sliding differences contrasts were again applied and restricted likelihood and Nelder–Mead optimisation were used. Like the main analysis, different models were again explored and the one with repetition as a fixed effect and a random intercept and random slope for participants was the winning model which was used for the following inferences. Response times were slowest for no repetition trials (M = 0.62, SD = 0.18; *b* = 1.76, SE = 0.04, *t* = 41.76, *p* < 0.001); the first repetition did not result in a benefit (M = 0.61, SD = 0.20; *b* = 0.02, SE = 0.02, *t* = 0.85, *p* = 0.41), but the second repetition did (M = 0.60, SD = 0.19; *b* = 0.09, SE = 0.03, *t* = 2.94, *p* = 0.007). There was no further benefit from three repetitions (M = 0.57, SD = 0.15, *b* = − 0.05, SE = 0.04, *t* = − 1.32, *p* = 0.19). This analysis suggests that participants were quicker at responding to the high prevalence target when it was presented on consecutive trials. However, the no repetition mean response time is still much lower than the low or medium prevalence conditions. Therefore, there is an effect of prevalence over and above the repetition effect.

### Discussion

Overall, the results of the current experiment further support the strong presence of the low prevalence effect as participants’ response times and accuracy were found to improve as target prevalence increased, irrespective of the reward assigned to targets. An exploratory analysis testing the effect of repeated presentation of high prevalence targets on participants’ performance, suggested that prevalence exerted an effect over and above the facilitation in response time that occurs from target repetitions. Experiment 4 included a temporal deadline and a reward, which were present in Experiment 3, allowing for a clearer assessment of the effect of unequal reward on prevalence. Overall, response times and miss rates were similar between Experiment 3 and Experiment 4. The notable decrease in response time and proportion of misses in the low prevalence condition of Experiment 3, compared to the low prevalence condition of Experiment 4, suggests that participants were more vigilant of low prevalence targets when those were associated with a higher reward compared with other targets versus when all targets were associated with the same reward level.

Exploratory analysis allowed for more detailed comparisons between the findings of the two experiments and the influence of unequal (Experiment 3) versus equal (Experiment 4) reward on the prevalence effect. This analysis confirmed that the effect of prevalence on response time and accuracy was stronger in Experiment 4 than in Experiment 3—the change in response time (Fig. 3, Panel A) and accuracy (Fig. 3, Panel B) between low and high levels of prevalence was more pronounced in Experiment 4. In addition, a follow-up analysis focusing just on Experiments 3 and 4, with ‘experiment’ as a between-subjects factor suggested a reliable interaction between prevalence and experiment. Given that the only difference between the two experiments was the nature of the reward distribution, this diminished effect of prevalence in Experiment 3 can only be attributed to the unequal reward distribution of the targets. However, it is worth noting that these are conclusions made from exploratory analysis and although they demonstrate a significant effect, stronger evidence can also be provided in a future investigation where unequal (Experiment 3) and equal (Experiment 4) reward distributions are directly compared in a single experiment.

One possible explanation for why participants did better (both in terms of response time and accuracy) in the low prevalence condition of Experiment 3 (with unequal reward) compared to the low prevalence of Experiment 4 (with equal reward) is the absolute point value associated with each condition. In particular, the total value of the low prevalence condition of Experiment 3 is 0.7 (0.1 prevalence * 7 points), whereas in the low prevalence condition of Experiment 4 is 0.2 (0.1 prevalence * 2 points). These findings indicate that reward can be used to attenuate the prevalence effect. It is likely that rewards are coded contextually, so that it is the relative rewards within a context that matter, rather than absolute values (e.g. Louie & Glimcher, 2012; Seymour & McClure, 2008). However, with our design we cannot differentiate between these possibilities and this could be a question to address in a future experiment.

## General discussion

Taken together, findings from the current experiments suggest that during MTS participants are able to prioritise search for specific targets in a graded manner based on their priority (prevalence in Experiments 1, 3 and 4; reward in Experiment 2). As priority increased participants were quicker and more accurate at correctly detecting a target, supporting past literature on relative prevalence (Godwin et al., 2010; Hout et al., 2015; Papesh et al., 2021; Walenchok et al., 2020) and reward (Gong et al., 2016; Kiss et al., 2009; Krebs et al., 2010; Serences, 2008)) manipulations. Additionally, when the two forms of priority (i.e. reward and prevalence), we observed a much stronger effect of prevalence on search performance compared to reward, as seen from the smallest effect coefficients of priority in Experiment 2 (i.e. reward manipulation) compared to all other experiments (i.e. where prevalence was manipulated as well; Fig. 3). This finding further supports previous studies in which prevalence had an overwhelming effect over and above reward (Clark & Gilchrist, 2018; Jiang et al., 2015; Wolfe et al., 2007; Won & Leber, 2016). However, unequal reward (Experiment 3) did not have a completely negligible effect on prevalence as, when compared to an equal reward (Experiment 4), it was found to diminish the prevalence effect, at least to a degree. The finding that prevalence had a weaker effect on visual search in Experiment 3 suggests that an unequal reward structure channelled some attention to low prevalence targets, which received the highest reward. Therefore, in the current MTS task, offering higher reward to low prevalence targets and lower reward to high prevalence targets weakened the prevalence effect, although not to the extent of causing its complete elimination or even reversal. However, the fact that an unequal reward distribution was not enough to overcome the low prevalence effect but was only able to diminish it suggests that in the current MTS task participants are more influenced by prevalence than reward information. These findings contradict previous investigations where researchers assigned a reward to targets in both high and low prevalence conditions and managed to counteract the rarity of low prevalence items (Navalpakkam et al., 2009, 2010). In such tasks, where single-target (i.e. a line bar) search was performed, equal reward was enough to eliminate the prevalence effect. In the current series of experiments, where participants had to remember and search for three different targets, reward might have not exerted a strong enough impact to eliminate the prevalence effect.

One potential explanation for the observed pattern of results regarding the stronger effect of prevalence on visual search performance, compared that of reward, might lie on the fact that presenting high prevalence targets more often than low prevalence targets improves the mental representation of the former over the latter, thereby facilitating detection of high prevalence targets. This stronger representation of the high over the low prevalence target results in the prioritisation of the former (irrespective of the higher reward associated with the latter). This suggestion is in line with principles of statistical learning during visual search, suggesting that patterns repeated often in our visual environment are extracted more easily than less repeated ones (Jones & Kaschak, 2012; Turk-Browne, 2012). Alternatively, given that participants were explicitly informed about the prevalence of different targets, it could be argued that they chose to prioritise search for the targets with the highest prevalence in order to improve their overall performance in the task and ensure that they would successfully find the target in the majority of trials. According to Clark and Gilchrist (2018) successfully finding a target during visual search task can be in itself rewarding, meaning that targets with a high probability of being presented are associated with a higher intrinsic reward (Wolfe, 2012b).

Additionally, one could argue that the failure of the unequal reward distribution in eliminating or reversing the low prevalence effect results from the fact that this reward pattern was not rewarding or motivating enough to dramatically increase participants’ vigilance for low prevalence targets and this can be seen as an important consideration of the this series of experiments. Therefore, future studies should explore different reward structures to investigate what level and distribution of reward may be able to reverse the prevalence effect. For instance, Navalpakkam et al. (2009) did not give the same reward to all types of errors as they employed what they refer to as an ‘Airport’ and ‘Gain’ feedback scheme that was found to be highly effective. According to this, participants lost more points for missing a target than for generating a false alarm and gained more points for correctly identifying a target than for correctly rejecting it. Given the positive impact that their reward scheme was found to have in reducing the prevalence effect, it is important to investigate it in a future visual search tasks with *multiple* targets as well, to see if this nullifying effect of reward on prevalence will continue to endure in a task with increased memory demands. Alternatively, Wolfe et al. (2018) also employed the method of both positive (receiving points for correctly detecting a target) and negative (losing points failing to detect a target) feedback in a hybrid foraging task; however, the total amount of points gained by participants was not turned into money but was instead used to determine when the task would be terminated. This meant that the higher the number of points received, the quicker the task would end. This was found to be an effective feedback pattern incorporating both reward and punishment, capable of eliminating the low prevalence effect in a hybrid foraging setting. It would be informative to explore a similar reward pattern in a future MTS experiment, to see whether using both reward and punishment and translating points gained into reduced task duration instead of money would be a more rewarding pattern sufficient to ameliorate the low prevalence effect to a larger extent than the reward pattern used in the current series of experiments.

Past literature findings have suggested that the visual search behaviour of participants is strongly mediated by expected value (Milstein & Dorris, 2007; Tobler et al., 2005). In particular, Navalpakkam et al. (2010) compared the impact of reward and prevalence in a complex perceptual environment where participants were searching for multiple targets. Participants’ visual search performance was equally guided by both value and salience consistent with a perfect (Bayesian) combination of both priority cues. If this was to be the case in the current task, then we would expect complete elimination of the low prevalence effect in Experiment 3 (but not any reversal of this effect), given that we purposely matched expected value across both high prevalence/low reward and low prevalence/high reward conditions (i.e. 0.7 × 1 = 0.7; 0.1 × 7 = 0.7, respectively). However, the stronger effect of prevalence over reward in the current study suggests that participants’ behaviour in this task is not guided by expected value associated with each target, contradicting past literature findings (Knutson et al., 2005). The current results suggest that different components of the expected value (i.e. probability and value) can be weighted differently in guiding search behaviour. Therefore, assuming that participants in the current series of experiments view different sources of priority (i.e. prevalence and value) differently, one possible option for future research would be to use a wider range of prevalence levels (e.g. 95%, 4% and 1%) and reward (e.g. 95 points, 4 points and 1 point, respectively). It could be the case that a reward will have a stronger impact in eliminating the prevalence effect when a higher reward value (e.g. 95 points) is associated with targets of much lower prevalence (i.e. 1%) which is something that warrants further investigation to draw more conclusive inferences about the impact of unequal reward on prevalence effect.

In the current series of MTS experiments, participants were explicitly informed about the prevalence associated with each of the three targets, in an attempt to resemble more closely real-life settings where participants during a visual search are often aware of the relative prevalence of the targets they are looking for (e.g. TSA agents during airport security screening are aware that knives, guns, and bombs are less prevalent targets in traveller’s luggage than bottles with liquids; doctors viewing medical X-rays are aware that tumours are less prevalent targets than soft tissue injuries). Research in judgement and decision-making suggests that information about event probabilities is treated differently by participants depending on how it is communicated and whether it is explicitly stated or implicitly learned through experience (Hertwig & Erev, 2009). Research findings regarding the explicit learning of prevalence information during an search task, and its impact on visual search performance, are not yet conclusive. For example, Ishibashi et al. (2012) and Lau and Huang (2010) found that the presence or absence of indicators regarding the likelihood of targets on each trial did not have a significant impact on the prevalence effect. Instead, observers relied more on their overall experience of target prevalence. Alternatively, Zhang and Houpt (2020) investigated how visual search performance of participants changes depending on whether they were informed explicitly about target prevalence or they learned it implicitly through experience on previous trials. Their results indicated a difference in these two conditions: participants were more biased towards giving a target-present response when searching for a high prevalent target in the condition where they were informed explicitly about its prevalence than when they inferred it through experience, while the opposite was true when searching for low prevalence targets. While there is a lot of literature on the prevalence effect in visual search, the impact of explicit knowledge of this prevalence on visual search warrants further investigation, as understanding how prevalence information is communicated and understood by observers, is critical in order to find ways to ameliorate this effect in important real-life contexts.

One important consideration for the current series of experiments is that the task and methodology used differ to a certain extent from real-life settings (e.g. airport security screenings, CCTV monitoring) where observers are searching for a high number of static targets at the same time (Wolfe, 2012a, 2012b). Therefore, an important next step in order to generalise our findings to the real-world contexts would be to investigate the impact of unequal reward distribution on the low prevalence effect during a multiple target search task where more than three targets were simultaneously searched for. Additionally, it is critical to investigate the impact of unequal reward distribution on the low prevalence effect during *categorical* search instead of a *target* search. In this case, the whole category of an item would be associated with different levels of prevalence and/or reward and not just individual targets. For example, participants could be searching for any bag, any pair of shoes or any teacup. This would offer a more ecological valid investigation of the prevalence effect in real-life settings as, for example, a TSA officer in the airport security screening is searching for any knife in the knife category and not for a specific knife. Therefore, it would be interesting to see if current findings regarding the elimination of the low prevalence effect using unequal reward distribution can also be generalised in a categorical search task.

Additionally, it is also important to consider the impact of on participants’ visual search performance, given that in the current paradigm distractors are the same for the whole experiment. With repeated exposure to distractors, participants become more familiar with them and may even develop mental templates of the distractors. This can result in reduced interference effects, more efficient distractor rejection and hence, facilitate search performance (Chun & Jiang, 1999; Endo & Takeda, 2004; Mruczek & Sheinberg, 2005). Hout and Goldinger (2010) found that during visual search tasks where both the target and distractors are real-world objects, the memory for the distractors is unintentionally formed. This incidental learning of distractors makes the search process more efficient as it leads to reduced search time (supporting Wolfe et al., 2002), with this effect being more pronounced when individuals are operating under a visual working memory load. The low prevalence effect may be attenuated or reduced when the same distractors are used repeatedly. Participants may become more adept at ignoring the distractors, allowing them to focus their attention more effectively on the search for the rare target. This familiarity-based improvement in distractor rejection would then counteract the negative impact of the prevalence effect, leading to faster and more accurate search performance. However, even if the low prevalence effect was attenuated to some extent by the repeated exposure to the same distractors in the current paradigm, it was clearly not abolished. Therefore, we can still assess the beneficial effects of unequal reward. It will be instructive to assess the interaction between prevalence and reward in settings with more unpredictable distractor items.

In our experimental design, a target (of any prevalence) was present on 50% of the total trials, and a low prevalent target was present on 10% of those target-present trials. This design is unlike other low prevalence experiments done in the past where a target, and specifically a low prevalence one, was present on only 1–2% of the trials (e.g. Wolfe et al., 2005, 2007). According to the Multiple Decision Model proposed by Wolfe and Van Wert (2010) responding 'target present' rarely in low prevalence search changes the overall search mechanism, leading to very different pattern of search (i.e. less accurate) as would be witnessed at high prevalence (see also Kunar et al., 2021). Therefore, it would be interesting to investigate the effect of unequal reward in an experiment where a target is present on only 1–2% of the trials. For example, it could be the case that because of the more often case of responding ‘target present’ in a task where a target is present on only 1–2% of the trials, a much higher reward would be need to diminish this effect.

An additional consideration for application to real-world circumstances would be that the method of reward is not a financially feasible strategy to be used for reducing the low prevalence effect in real-life settings like airport security screenings and medical x-rays. However, given the robustness of the prevalence effect and how difficult it is to overcome it (Wolfe et al., 2007), it is important to acknowledge that reward is one of the few methods which has promising impact on this effect (i.e. current findings, Navalpakkam et al., 2010; for other methods see also Kunar et al., 2021; Taylor et al., 2021) and could therefore be considered at least during training phase of observers in the aforementioned contexts, where they will be assigned a larger amount of credits or bonus points for detecting low prevalence targets and a lower amount of credits or points for detecting high prevalence targets. In this way, observers’ sensitivity to low prevalence targets might be increased causing them to respond more quickly and accurately to the targets with the higher importance for the health and safety of the public. In particular, it has been found that beneficial effects of monetary reward on attentional capture during visual search persist even when reward is removed in the actual testing phase (Lee et al., 2022; Watson et al., 2019).

The aim of the current experiment was to investigate whether unequal reward can be used to modulate the low prevalence effect in a visual search task with multiple targets. The findings of the current series of experiments suggest that participants are able to prioritise search for some targets versus others based on their assigned priority (i.e. reward or prevalence). Nevertheless, when the two types of priority were combined, results indicated a stronger effect of prevalence over reward. Two different types of reward distribution were used, equal and unequal, with findings suggesting that neither of the reward schemes was able to eliminate, let alone reverse, the robust effect of prevalence. However, the unequal reward distribution was able to diminish the effect of prevalence on visual search to a certain degree as indicated by faster response times and fewer misses in the low prevalence condition, compared to the equal reward condition. Current results offer a unique contribution to the literature as the interaction of two of the strongest impact factors on visual search (i.e. prevalence and reward) is investigated in a task where more than two targets are simultaneously searched for, something which has largely been overlooked in the literature as the majority of visual search experiments investigating the interaction of reward and prevalence only ever used (mostly) one or (rarely) two targets (Ort & Olivers, 2020).

## Data Availability

Each of the experiments was pre-registered on the Open Science Framework. Pre-registration information, the code for running the experiments, data and analysis scripts as well as stimuli used can be found on the OSF (Experiment 1: https://osf.io/cbueg/; Experiment 2: https://osf.io/gnjbx/; Experiment 3: https://osf.io/hrftb/; Experiment 4: https://osf.io/a3729/).; Experiment 3: https://osf.io/hrftb/; Experiment 4: https://osf.io/a3729/). Authors declare no conflict of interest.

## Footnotes

1 The values of prevalence and reward were chosen based on past studies of the interaction between reward and prevalence (Clark & Gilchrist, 2018; Wolfe et al., 2018; Won & Lever, 2016). Lower values have also been used in typical prevalence experiment (e.g. low prevalence target being presented only on 1% or 2% of trials; Wolfe et al., 2005; Wolfe et al., 2007), which is something further elaborated in the discussion.

2 Details of the four statistical models considered were not included in the pre-registration of the four Experiments. The authors chose to include in the pre-registration an outline of the general analysis plan regarding the dependent variables of interest, response time and accuracy (i.e. LME and multiple-logistic regression, respectively). The approach taken for model specification was to explore models from minimal (null effects) to maximal (random intercepts and slopes; Barr et al., 2013). It was an oversight not to include this approach in the pre-registration, however, which of these models would be eventually chosen to draw inferences from could not have been pre-determined as this was dependent on the data and which of the models explored gave the best fit.

3 Data were also analysed aggregated across trials (as is more typical of previous studies in this field), and similar effects are observed across all four Experiments.

4 The number of trials changed compared to the other experiments of this series (Experiments 1, 3 and 4 where 400 experimental trials, 200 target-present and 200 target absent, were included in each) because an equal number of high-reward, middle-reward and low-reward had to be included (as only reward was manipulated and prevalence of targets in the target-present trials had to remain constant). If 400 trials were included like in the other experiments, with 200 target-present and 200 target-absent trials, an equal number of trials across the three reward levels would not have been possible (i.e. the quotient of 200 by 3 is not a whole number).

5 It is worth noting that in Experiments 3 and 4 a few participants exhibited a higher proportion of errors in the low prevalence condition. Just to ensure that this did not impact the overall effect, analysis was also performed excluding those participants and a similar pattern of results was observed. However, because this high proportion of errors is attributed to the low prevalence of the target and is therefore considered a result of the experimental manipulation, in the analysis reported here participants were not excluded based on their proportion of errors (only on their response times, as explained in text).
